# Paediatric computed tomography and subsequent risk of leukaemia, intracranial malignancy and lymphoma: a nationwide population-based cohort study

**DOI:** 10.1038/s41598-020-64805-8

**Published:** 2020-05-08

**Authors:** I-Gung Li, Yao-Hsu Yang, Yiu-Tai Li, Yuan-Hsiung Tsai

**Affiliations:** 1 Department of Diagnostic Radiology, Chang Gung Memorial Hospital, Chiayi County, Taiwan; 2 Health Information and Epidemiology Laboratory, Chang Gung Memorial Hospital, Chiayi County, Taiwan; 3 Department of Traditional Chinese Medicine, Chang Gung Memorial Hospital, Chiayi County, Taiwan; 4 School of Traditional Chinese Medicine, College of Medicine, Chang Gung University, Taoyuan, Taiwan; 5 Department of Obstetrics and Gynecology, Kuo General Hospital, Tainan, Taiwan

**Keywords:** Cancer prevention, Risk factors

## Abstract

Red bone marrow and brain tissue are highly radiosensitive in children. We investigate the relationship between childhood computed tomography (CT) exposure and leukaemia, intracranial malignancy and lymphoma. All participants in the study were aged less than 16 years. A total of 1,479 patients in the leukaemia group, 976 patients in the intracranial malignancy group and 301 patients in the lymphoma group were extracted from the Catastrophic Illness Certificate Database in Taiwan as the disease group. In total, 126,677 subjects were extracted from the Longitudinal Health Insurance Database 2010 of the Taiwan National Health Insurance Research Database as the non-disease group. The odds ratios (ORs) and 95% confidence intervals (CIs) for childhood CT exposure and times of childhood CT were estimated. Childhood CT exposure was correlated to the intracranial malignancy group in both one-year (OR = 1.95, 95% CI 1.40–2.71, *p* < 0.001) and two-year (OR = 1.56, 95% CI 1.04–2.33, *p* = 0.031) exclusion periods. The time of childhood CT was also correlated to intracranial malignancy in both one-year (OR = 1.69, 95% CI 1.34–2.13, *p* < 0.001) and two-year (OR = 1.55, 95% CI 1.17–2.04, *p* = 0.002) exclusion periods. The results indicated that childhood CT exposure was correlated with an increased risk of future intracranial malignancy.

## Introduction

Computed tomography (CT) is widely used in modern hospitals and provides accurate, timely information. A previous study estimated that 29,000 cases of cancer and 14,500 deaths in the general population annually in the U.S. may be induced by CT exposure^1^. An official report from Australia noted that an irradiation dose of over 20 mSv for the general population was associated with a greater than one in 1000 risk of developing fatal cancers, non-fatal cancers and serious hereditary diseases^2,3^. Recently, many studies have found that ionizing radiation exposure in children may cause serious complications, including malignancy^4–7^. Several studies demonstrated that red bone marrow and brain tissue were highly radiosensitive, especially during childhood^8–10^. A recent report pointed out that the increase in the incidence of all cancers in CT-exposed individuals aged 0–19 years was 9.38 per 100,000 person years in Australia^5^. A study from the United Kingdom reported that the relative risk (RR) for individuals aged 0–22 years who received bone marrow radiation doses greater than 30 mGy was 3.18, and the risk for patients who received brain radiation doses greater than 50 mGy was 2.82^4^.

Leukaemia, intracranial malignancy and lymphoma are the most common malignancies in children in Taiwan^11^. Many studies have pointed out a relationship between leukaemia, intracranial malignancy and ionizing radiation exposure^1,9,12–16^. Therefore, we aim to investigate the relationships between childhood CT exposure and these three major malignancies using cohort data from the Taiwan National Health Insurance research database (NHIRD).

## Results

Data from 126,677 subjects were extracted from the Longitudinal Health Insurance Database (LHID) 2010, and 2,202 subjects were excluded due to either cancer or any nuclear medicine procedure history. Therefore, a total of 124,475 subjects were in the non-disease group and were matched to each disease group as controls. In the leukaemia group, 1,583 patients with leukaemia were recruited from the Catastrophic Illness Certificate Database (CICD). Among them, 57 patients were excluded due to either other cancer or any nuclear medicine procedure one year before the diagnosis of leukaemia. A total of 1,423 leukaemia patients in the final leukaemia group were matched to 14,230 subjects from the non-disease group for a ratio of one leukaemia case to ten controls matched by gender, year of birth and urbanization level. In the intracranial malignancy group, 897 patients with intracranial malignancy were recruited from the CICD. Among them, 37 patients were excluded due to either other cancer or any nuclear medicine procedure one year before the diagnosis of intracranial malignancy. A total of 838 patients in the final intracranial malignancy group were matched to 8,380 subjects from the non-disease group for a ratio of one intracranial malignancy case to ten controls matched by gender, year of birth and urbanization level. In the lymphoma group, 301 patients with lymphoma were recruited from the CICD. Among them, 21 patients were excluded due to either other cancer or any nuclear medicine procedure one year before the diagnosis of lymphoma. A total of 272 patients were included in the final lymphoma group and were matched to 2,720 subjects from the non-disease group for a ratio of one lymphoma case to ten controls matched by gender, year of birth and urbanization level.

The characteristics of the study subjects in this article are shown in Table 1. The adjusted ORs of childhood CT exposure and times of childhood CT in one- and two-year exclusion periods are listed in Table 2. The adjusted ORs of childhood CT exposure were significantly increased in the cases of the intracranial malignancy group compared to the controls in both one-year (OR = 1.95, 95% CI 1.40–2.71, *p* < 0.001) and two-year exclusion periods (OR = 1.56, 95% CI 1.04–2.33, *p* = 0.031). The adjusted ORs of times of childhood CT were also significantly increased in both one-year (OR = 1.69, 95% CI 1.34–2.13, *p* < 0.001) and two-year exclusion periods (OR = 1.55, 95% CI 1.17–2.04, *p* = 0.002) compared to controls. The adjusted ORs of greater or equal to two times of childhood CT were significantly increased in both one-year exclusion period (OR = 2.80, 95% CI 1.21–6.48, *p* = 0.001) and two-year exclusion periods (OR = 2.84, 95% CI 1.05–7.69, *p* = 0.040) compared to controls. The adjusted ORs showed no significant difference for childhood CT exposure and times of childhood CT in both the leukaemia and the lymphoma group. Considering that the age at first CT exposure may affect the occurrence of childhood leukaemia, intracranial malignancy and lymphoma, an analysis was performed, and the results are listed in Table 3. The results showed no significant difference in age at first CT exposure between those younger and equal to six years old and those older than six years old in both one- and two-year exclusion periods in the three groups.

**Table 1:** Characteristics of the Study Patients with Leukaemia, Intracranial Malignancy and Lymphoma from the CICD and of the Comparison Controls from the LHID 2010.

Variables	Leukaemia		Control		*p* value	Intracranial malignancy		Control		*p* value	Lymphoma		Control		*p* value
	(*n* = 1423)		(*n* = 14230)			(*n* = 838)		(*n* = 8380)			(*n* = 272)		(*n* = 2720)		
	*n*	%	*n*	%		*n*	%	*n*	%		*n*	%	*n*	%	
Gender					1.000					1.000					1.000
Male	791	55.6	7910	55.6		479	57.2	4790	57.3		169	62.1	1690	62.1	
Female	632	44.4	6320	44.4		359	42.8	3590	42.7		103	37.9	1030	37.9	
Age on index day*					1.000					1.000					1.000
≤6	1103	77.5	11030	77.5		542	64.7	5420	64.7		115	42.3	1150	42.3	
>6	320	22.5	3200	22.5		296	35.3	2960	35.3		157	57.7	1570	57.7	
Urbanization level					1.000					1.000					1.000
1 (city)	400	28.1	4000	28.1		217	25.9	2170	25.9		76	27.9	760	27.9	
2	701	49.3	7010	49.3		378	45.1	3780	45.1		120	44.1	1200	44.1	
3	207	14.6	2070	14.6		147	17.5	1470	17.5		40	14.7	400	14.7	
4 (villages)	115	8.1	1150	8.1		96	11.5	960	11.5		36	13.2	360	13.2	
CT: one-year exclusion period					0.853					<0.001					0.247
Yes	34	2.4	329	2.3		45	5.4	240	2.9		13	4.8	93	3.4	
No	1389	97.6	13901	97.7		793	94.6	8140	97.1		259	95.2	2627	96.6	
CT: two-year exclusion period					0.494					0.031					0.860
Yes	21	1.5	245	1.7		29	3.5	190	2.3		7	2.6	75	2.8	
No	1402	98.5	13985	98.3		809	96.5	8190	97.7		265	97.4	2645	97.2	

Abbreviations: LHID = Longitudinal Health Insurance Database; CICD = Catastrophic Illness Certificate Database; CT = computed tomography.

*The index date was the disease diagnosis date in the disease group and the corresponding date in the non-diseased group.

**Table 2:** The Adjusted ORs for CT Exposure and Its Frequency for Leukaemia, Intracranial Malignancy and Lymphoma with One-year or Two-year Exclusion Period.

Variables	Leukaemia		Intracranial malignancy		Lymphoma	
	Adjusted OR*	*p* value	Adjusted OR*	*p* value	Adjusted OR*	*p* value
**One-year exclusion period**						
CT						
No	1.00		1.00		1.00	
Yes	1.04(0.72–1.48)	0.851	1.95(1.40–2.71)	<0.001	1.42(0.78–2.59)	0.246
Times of CT	1.14(0.89–1.47)	0.293	1.69(1.34–2.13)	<0.001	1.33(0.82–2.15)	0.243
Times of CT/cases						
0	1.00		1.00		1.00	
1	0.96(0.65–1.43)	0.853	1.84(1.29–2.63)	0.001	1.35(0.71–2.57)	0.364
≥2	1.59(0.67–3.76)	0.295	2.80(1.21–6.48)	0.016	2.03(0.44–9.33)	0.362
**Two-year exclusion period**						
CT						
No	1.00		1.00		1.00	
Yes	0.85(0.54–1.34)	0.492	1.56(1.04–2.33)	0.031	0.93(0.42–2.05)	0.859
Times of CT	1.07(0.78–1.45)	0.686	1.55(1.17–2.04)	0.002	0.96(0.50–1.86)	0.907
Times of CT/cases						
0	1.00		1.00		1.00	
1	0.74(0.44–1.23)	0.246	1.43(0.92–2.21)	0.113	0.88(0.38–2.05)	0.767
≥2	1.72(0.66–4.45)	0.266	2.84(1.05–7.69)	0.040	1.43(0.18–11.67)	0.740

Abbreviations: OR = odds ratio; CT = computed tomography.

*The model was adjusted by year of birth, gender and urbanization level.

**Table 3:** The Adjusted ORs for the Age at First CT Exposure for Leukaemia, Intracranial Malignancy, and Lymphoma in One-year or Two-year Exclusion Period.

Variables	Leukaemia			Intracranial malignancy			Lymphoma		
	*n*	Adjusted OR*	*p* value	*n*	Adjusted OR*	*p* value	*n*	Adjusted OR*	*p* value
**One-year exclusion period**									
Age at first CT exposure									
≤6-year-old	30	1		37	1		10	1	
>6-year-old	4	0.91(0.31–2.72)	0.868	8	1.22(0.53–2.84)	0.638	3	1.17(0.29–4.67)	0.826
**Two-year exclusion period**									
Age at first CT exposure									
≤6-year-old	20	1		24	1		6	1	
>6-year-old	1	0.40(0.05–3.13)	0.386	5	1.59(0.55–4.60)	0.391	1	0.73(0.08–6.53)	0.776

Abbreviations: OR = odds ratio; CT = computed tomography.

*The model was adjusted by year of birth, gender and urbanization level.

## Discussion

This study is a nationwide, population-based, retrospective, case-control study. In this study, for intracranial malignancy patients under 16 years of age, childhood CT exposure resulted in significantly elevated ORs compared to controls in both the one- and two-year exclusion periods. The times of CT also significantly correlated with the risk of intracranial malignancy in both one- and two-year exclusion periods. The results indicated that childhood CT exposure was correlated with increasing risk of a future intracranial malignancy in Taiwan; however, the same trend was not found in the leukaemia or lymphoma group. A current theory postulates that ionizing radiation energy overpowers the binding energy of atom-orbiting electrons and knocks electrons out of their orbit, thereby creating radicals. Biological effects cause double-strand DNA breaks or damage, and cancer may be induced by occasional point mutations, chromosome translocations or gene fusion^16^.

An investigation of radiation-induced cancer in atomic bomb survivors found that leukaemia developed in the majority of children; most types of leukaemia, other than chronic lymphocytic leukaemia, may be induced by ionizing radiation with a minimum latency of approximately two years^17^. Some reports also mentioned that childhood leukaemia was diagnosed 3 years after the first exposure; however, the peak diagnosis time was 6–8 years^18–20^. Although leukaemia is strongly associated with childhood radiation exposure in atomic bomb survivors, a similar result was not established in this study. Ionizing radiation has been found to induce some types of nervous system tumours and shows marked differences depending on age at exposure. The United Nations Scientific Committee on the Effects of Atomic Radiation (UNSCEAR) 2006 Report demonstrated that malignant tumours of the central nervous system were reported mostly after high-dose radiotherapy and after exposure in childhood. Gliomas are the tumours with the highest risk in those younger than five years old exposed to radiation; this risk dramatically decreases in those older than 20 years old, possibly indicating that susceptibility markedly decreases as brain development nears completion^21^.

Previous studies about the correlations between childhood CT exposure and the risk of lymphoma, leukemia and brain tumors are summarized in Table 4. Some causal links between childhood CT exposure and intracranial tumour incidence were identified with similar results in previous studies. According to an investigation of the cancer incidence rate of 10.9 million people in Australia, the overall incidence rate ratio (IRR) for brain cancers (ICD-10: C69–72) based on a one-year exclusion period was 2.13 (CI 1.88–2.41)^5^. A UK study found that the excess relative risk (ERR) per mGy for brain tumours in a 5-year exclusion period was 0.023 (95% CI 0.010–0.049; *p* < 0.0001). The relative risk of brain tumour for individuals who received a cumulative dose of 50–74 mGy (mean dose 60.42 mGy) was up to 2.82 (95 CI: 1.33–6.03) compared with individuals who received a dose of less than 5 mGy. For individuals who received a cumulative dose of 50 mGy or more (mean dose 104.16 mGy), the relative risk was 3.32 (95 CI: 1.84–6.42)^4^. A German study also reported a standardized incidence ratio (SIR = cancer observed/expected) of 1.35 (95% CI 0.54–2.78) for CNS tumours^22^. Another study including data from 21 French university hospitals based on a one-year exclusion period reported an ERR per mGy of 0.017 (95% CI 0.010–0.044) for CNS cancer. After adjusting for known predisposing factors, the authors observed no significant increased risk related to CT exposure^23^. A Taiwanese study also reported that the hazard ratio (HR) for all brain tumours was higher, at 2.56 (95 CI 1.44–4.54, *p* < 0.01), in the exposed cohort than in the unexposed cohort, but the overall risk of malignancy and benign brain tumour was not significantly different between the two cohorts. The frequency of CT scans showed a strong correlation with all brain tumours (increase in HR from 2.32 to 10.4, *p* = 0.0001) compared with the unexposed cohort^24^, similar to the finding in our study.

**Table 4:** Comparison of Epidemiology and Risk Evaluations for Leukaemia, Brain Tumour and Lymphoma with our Study.

	Exclusion period (Year)	Database	Sample size	Age	Follow up period	Leukaemia result	Brain tumour result	Lymphoma result
Pearce *et al*. (2012 retrospective cohort study)	2 for Leukaemia; 5 for brain tumour	NHS, United Kingdom	178,604 with Leukaemia; 176587 with brain tumour	0–22	1985–2008	ERR per mGy: 0.036 (95% CI 0.005–0120; *p* = 0.0097)	ERR per mGy: 0.023 (95% CI 0.010–0.049; *p* < 0.0001)	NA
Mathews *et al*. (2013 retrospective cohort study)	1	AIHW, Australia	10,939,680	0–20	1985–2007	Leukaemias and myelodysplasias IRR: 1.23 (95% CI: 1.08–2.41) ERR per mGy: 0.039 (95% CI: −0.014–0.070)	All CT scan: IRR: 2.13 (95% CI: 1.88–2.41); Only Brain CT: IRR: 2.44 (95% CI: 2.12–2.81) ERR per mGy: 0.029 (95% CI: −0.023–0.037)	Hodgkin’s lymphoma IRR: 1.15 (CI:1.01–1.32); Other lymphoma IRR: 1.01 (CI: 0.82–1.23)
Huang *et al*. (2014 retrospective cohort study)	2	NHIRD, Taiwan	122,086	0–18	1998–2008	HR: 1.90 (95% CI: 0.82–4.40)	All brain tumour HR: 2.56 (95% CI: 1.44–4.54; *p* < 0.01); Malignancy HR: 1.84 (95% CI: 0.64–5.29)	NA
Journy *et al*. (2015 retrospective cohort study)	1	23 department, France	67,274	0–10	2000–2010	ERR per mGy: 0.014 (95% CI: −0.037–0.065)	ERR per mGy: 0.017 (95% CI: −0.010–0.044)	ERR: −0.002 (95% CI: −0.050–0.046)
	2					ERR per mGy: 0.047 (95% CI: −0.065–0.159)	ERR per mGy: 0.012 (95% CI: −0.013–0.037)	ERR: 0.008 (95% CI: −0.057–0.073)
Krille *et al*. (2015 retrospective cohort study)	2	GCCR, Germany	44,584	0–15	1980–2010	SIR: 1.72 (95% CI: 0.89–3.01)	SIR: 1.35 (95% CI: 0.54–2.78)	SIR: 3.26 (95% CI: 1.63–5.83)
Li *et al*. (2020 retrospective cohort study)	1	NHIRD, Taiwan	126,677	0–16	1998–2013	OR: 1.04(95% CI: 0.72–1.48)	OR: 1.95 (95% CI:1.40–2.71, P < 0.001)	OR: 1.42 (95% CI: 0.78–2.59)
	2					OR: 0.85 (95% CI: 0.54–1.34)	OR: 1.56 (95% CI:1.04–2.33, P = 0.031)	OR: 0.93 (95% CI: 0.42–2.05)

Abbreviations: NHS = National Health Service; AIHW = Australian Institute of Health and Welfare; NHIRD = National Institutes of Health research database;

GCCR = German Childhood Cancer Registry; RR = risk ratio; HR = hazard ratio; ERR = excess relative risks; OR = odds ratio; SIR = standardized incidence ratios; NA = not available; 95% CI = 95% confidence interval.

However, the evidence linking childhood lymphoma and exposure to ionizing radiation is weak. According to the UNSCEAR 2013 report, Hodgkin lymphomas were barely associated with ionizing radiation^17^. For non-Hodgkin lymphomas, the Life Span Study reported no association with ionizing radiation in males, but there was an association in females^25^. Overall, there is no current evidence for a significant dose-response relationship for either Hodgkin or non-Hodgkin lymphomas, similar to the finding in our study. Regarding the age at exposure, a previous report on atomic bomb exposure survivors^26^ noted that the younger population may have higher risk for malignancy. However, we did not find the same trend in our study. A small sample size and uneven sample size distribution may cause this result.

The reason for using an exclusion period in this study, as well as in the previous literature, was to exclude the possibility of reverse causation because either precancerous or early-stage symptoms of cancer itself might prompt a CT scan, potentially causing indication bias^27^. The exclusion period before the disease diagnosis is critical, and both one and two years were included in this study. An applicable length of the exclusion period between the exposure to ionizing radiation and the progression of associated cancers is unclear^24^. Some studies demonstrated that leukaemia cases occurred as early as 1–3 years after radiation therapy of cancer^28–30^. A 2014 report from the Centers for Disease Control and Prevention (CDC) suggests that the minimum exclusion period for lymphoproliferative and haematopoietic cancers (including all types of leukaemia and lymphoma) is 0.4 years, and the minimum period for childhood cancers (other than lymphoproliferative and haematopoietic cancers), including brain tumours, is 1 year^31^. Unfortunately, the current data pool with longer exclusion periods is not large enough, and a large-scale study with a longer follow-up period is necessary to confirm this result.

This study had several strengths. A large sample size was obtained with the use of nationwide data, resulting in increased statistical sensitivity and power. The large sample size also minimized the loss to follow-up and allowed the accurate calculation times of CT in all subjects due to the widespread coverage of the NHI database, which included more than 99% of the population in Taiwan. Each leukaemia, brain malignancy and lymphoma diagnosis was made by a physician and reported with ICD-9-CM codes in the Registry of Catastrophic Illnesses patient database. Misclassification bias was minimized due to the strict censoring of this social welfare database.

This study also had some limitations. First, uniformly estimated organ-absorbed radiation doses to evaluate the dose-response relationship were not achieved because of the lack of information on actual absorbed doses from NHIRD. Consequently, we adopted childhood CT exposure and times of childhood CT exposure as our target variables due to their applicability to real life, and we believed that a large sample size might mediate the large variations in effective radiation doses. The results of our study are in line with previous reports that both the times and radiation dose of CT exposure are related to childhood malignancies^4–6,22,24,27^. Second, basic medical information and risk factors other than CT exposure that might associate with the development of malignancies were not included in the analysis. We did check the comorbidities, including some rare diseases associated with malignancies for each subject, but only few cases were identified and thus were ignored in the analysis. Information regarding other risk factors such as environment and substance exposure were not included in the database. Furthermore, this study did not consider radiation doses from other common medical examinations. In addition to CT, many other iatrogenic ionizing radiation examinations, including conventional X-ray imaging and intervention procedures, are administered in the hospital. The CT ionizing radiation doses are 100 to 500 times higher than those of conventional radiography and are more likely to be linked to an increased risk of cancer^32,33^.

## Methods

### Data source

#### Longitudinal Health Insurance Database (LHID) 2010

LHID 2010 is a subset of data that contains the original claims data of 1 million beneficiaries systemically and randomly sampled from the year 2010 Registry for Beneficiaries of the NHIRD. Everyone in the year 2010 Registry for Beneficiaries of the NHIRD was a beneficiary of the National Health Insurance program during the period of Jan. 1, 2010 to Dec. 31, 2010 and was drawn for random sampling. The latest version of LHID 2010 includes the medical claims of all beneficiaries during the period of 1997 and 2013. This database has been widely applied in epidemiologic and medical research and contains information on prescription use, diagnoses, and cost of hospitalizations^34^. The Taiwan National Health Insurance (NHI) reimbursement system has been in use since March 1995 and contains de-identified medical claims from 98% of the population of Taiwan (23 million people). The detailed medical claims include recorded outpatient visits, hospital admissions, prescriptions, and procedures including times of CT and diagnosis of disease based on International Classification of Diseases, Ninth Revision, Clinical Modification (ICD-9-CM) code. The NHIRD is anonymized and maintained by the National Health Research Institute with confidentiality according to the Personal Electronic Data Protection Law. There were no significant differences in age, gender and average premium rate between individuals in the LHID 2010 and those in the original NHIRD^35^.

#### The Catastrophic Illness Certificate Database (CICD)

Under the NHI program, patients with severe illnesses can apply for catastrophic illness certification, and those who receive care for their illness or related conditions within the certificate’s validity period do not pay a co-payment for outpatient or inpatient care. Patients with such illnesses are exempt from medical costs; therefore, the registry database is comprehensive and has excellent validity. The CICD was used to identify cancer patients by ICD-9-CM codes; all cancer patients were histologically or cytologically confirmed before a catastrophic illness certificate was issued during the period of 1997 through 2013.

### Population

**Figure 1:**
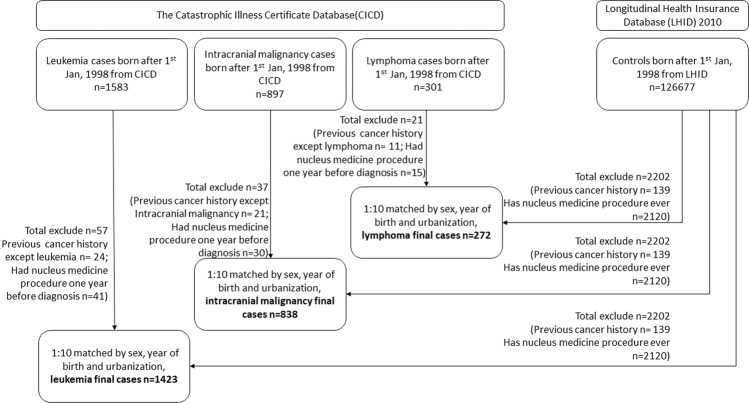
Flowchart for selecting the study participants. Abbreviations: LHID = Longitudinal Health Insurance Database; CICD = Catastrophic Illness Certificate Database; ICD-9 = International Classification of Disease Ninth Revision.

Figure 1 shows the flowchart for selecting the participants in this study. All recruited individuals in this study were only born after Jan. 1, 1998 to ensure that all of them were under 16 years of age throughout the whole investigational period and complete medical records were available in LHID 2010 and CICD during the period 1997 through 2013. Children who had leukaemia, intracranial malignancy or lymphoma were selected by ICD-9-CM code (leukaemia 204–208.91; intracranial malignancy 191–192.9, 194.3–194.4; lymphoma 200–202.28, 2028–202.98) from CICD during the period from 1998 through 2013 as the disease groups. In these three main disease groups, those who had other cancers (ICD-9-CM code 140-208.91; other than 204-208.91 in leukaemia cases; 191–192.9, 194.3–194.4 in intracranial malignancy cases and 200–202.28, 2028–202.98 in lymphoma cases) or nuclear medicine procedures one year before disease diagnosis were excluded. Patients who were unable to be matched to the non-disease group were not enrolled in the final disease group. Individuals were recruited from the LHID 2010 as the non-disease group, and those who had cancer history (ICD-9-CM code 140-208.91) or any nuclear medicine procedure were also excluded.

### Exposure and statistical analysis

**Figure 2:**
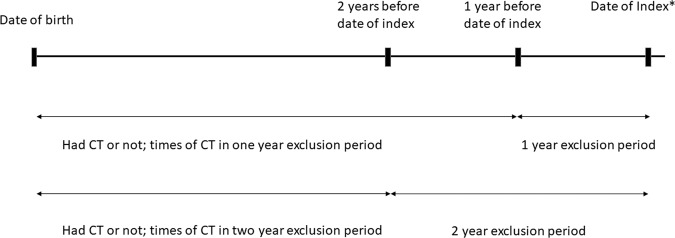
Study design. This figure shows the investigation of childhood CT exposure and times of childhood CT in case-control matched subjects. The index date was the disease diagnosis date in the disease group and the corresponding date in non-diseased group.

For each individual in the disease group, ten non-diseased individuals as controls were matched by year of birth, gender and urbanization level. Figure 2 shows the study design in this article. Childhood CT exposure and times of childhood CT one and two years before the index date were recorded. Any CT scan within one and two years before the index date was ignored. This exclusion period was established due to the possibility that CT scan was part of the cancer diagnostic procedure. The index date was the disease diagnosis date in the disease group and the corresponding date in the non-diseased group. The distributions of gender, year of birth, urbanization level and times of CT scans between the cases and the controls were compared. Logistic regression was performed to calculate the odds ratios (ORs) and 95% confidence intervals (CIs) for childhood CT exposure and times of childhood CT between cases and controls in these three main disease groups with adjustments for birth year, gender and urbanization level. Logistic regression was also performed to calculate the odds ratios (ORs) and 95% confidence intervals (CIs) of the age of first CT exposure between patients younger and equal to six years old and older than six years old in disease groups. A two-tailed *p* value of <0.05 was considered statistically significant. All statistical analyses in this study were performed using SAS statistical software (version 9.4 for Windows; SAS Institute, Inc., Cary, NC, USA). This study was approved by the Institutional Review Board (IRB) of Chang Gung Medical Foundation (No. 201700967B0), which approved the experiments, including any relevant details that were performed in accordance with relevant guidelines and regulations. In our study, de-identification was performed for the whole manuscript, and the IRB approved a waiver of the informed consent form.

## Data Availability

All data in this article are available from the National Health Insurance Research Database (NHIRD) published by the Taiwan National Health Insurance (NHI) Bureau. Due to legal restrictions imposed by the government of Taiwan, data cannot be made publicly available. Data requests can be performed by formal proposal to the NHIRD (http://nhird.nhri.org.tw).
